# Sphingolipids, mycobacteria and host: Unraveling the tug of war

**DOI:** 10.3389/fimmu.2022.1003384

**Published:** 2022-09-15

**Authors:** Shakeel Ahmed Mohammed, Reena Vohra Saini, Abhimanyu Kumar Jha, Vijay Hadda, Amit Kumar Singh, Hridayesh Prakash

**Affiliations:** ^1^ Amity Institute of Virology and Immunology, Amity University, Noida, India; ^2^ Department of Biotechnology, Maharishi Markandeshwar (M. M). Engineering College, Maharishi Markandeshwar (Deemed to be University), Ambala, India; ^3^ Department of Biotechnology, Sharda University, Greater Noida, India; ^4^ Department of Pulmonary Medicine and Sleep Disorders, All India Institute of Medical Sciences, New Delhi, India; ^5^ Experimental Animal Facility, National JALMA Institute for Leprosy and Other Mycobacterial Diseases, Tajganj Agra, India

**Keywords:** Sphingosine-1phosphate, M1 effector macrophages, iNOS, tuberculosis, innate immunity significance

Mycobacteria exploit sphingolipid’s metabolism for their opportunistic survival which is evident with compromised sphingolipid metabolism in TB patients. Therefore, regulating sphingolipid metabolism seems to be one of the novel modalities for controlling the disease. Several studies including our pioneer studies potentially identified sphingolipids as one of the active constituents of metabolism having anti-mycobacterial efficacy both in macrophages and whole organisms. On the basis of this and our preliminary data, we feel that adjusting sphingolipids is a prudent approach for managing latent TB as well and is anticipated to be contributing significantly to TB-related death. This concept holds tremendous therapeutic potential and is believed to contribute significantly to hostdirected therapy against Tuberculosis. Sphingolipids are a dual-specific category of lipids that can favor both host and pathogens depending upon their metabolism which is interconvertible and have an influence on host immunological response against infection. Sphingolipids are active constituents of mucus, and lipids raft and account for 10-20% of total cellular lipids. These are decisive for maintaining membrane integrity as well as cells to cell communication (1). Sphingolipids actively participate in a plethora of cellular processes like secretion, endocytosis, chemotaxis, inflammation and cellular homeostasis (1–5). Disturbance in sphingolipid metabolism is associated with various respiratory, neural, and immune-mediated diseases. Among various sphingolipid derivatives which have been identified so far, sphingosine-1-phosphate (S-1P) and ceramide-1 phosphate are the best-studied sphingolipid derivatives biologically. S-1P is a secondary messenger and one of the best-explored sphingolipid moiety (4) and is involved in a plethora of biological responses like immune cell trafficking (6), vascular permeability (7), cellular differentiation (8), etc. S-1P signal *via* G protein-coupled receptors (GPCRs) coupled receptors known as S-1PR1-5 and mediates pleiotropic impact of S-1P. Some key studies have shown that S-1P activates calcium signaling (9) enhance phospholipase D activity (10), and induce respiratory burst in alveolar macrophages (11) indicating its immune-modulatory potential.

For last couples of years, we have exploited the impact of sphingolipids (mainly S-1P) on innate immune defense mechanism against tuberculosis infection (12, 13). In view of this here we will summarize the significance of S-1P on protective immunity against *Mycobacterium tuberculosis* infection in host. In this context, previous report (13, 14) and recent inclusion by our group (12) have demonstrated anti-mycobacterial potential of S-1P in animal model system. Of particular interest, our recent study clearly demonstrated the impact of S-1P on activation of inducible Nitric Oxide Synthase (iNOS) proteins in macrophages and their subsequent differentiation toward M1 effector phenotype as well as increased infiltration of S1PR3+ CD11b+ alveolar macrophages during animal challenge study with *M.tb (* 12). Most interestingly, our study suggested a possible crosstalk of S-1P with interferon gamma (IFN-ү) in infected animals during infection (12) accounting for S-1P mediated protection of animal from *M. tuberculosis* infection.

On the basis of these compelling data, we here propose that exploiting S-1P seems to be a prudent approach for controlling TB burden in clinics. This is particularly relevant because TB patients are poor in their sphingolipid metabolites like S-1P which is due to utilization of this enzymes for encountering stress in macrophages (15). Therefore, supplementing TB patients with S-1P for the management of pulmonary TB warrants its clinical application. However due to allergic and autoimmune manifestation of S-1P, it may not qualify pharmacological criteria to be included in current TB regimen at the moment. Therefore, enhancing S-1P contents either *via de novo* or salvage pathways becomes essential. In this context, adapting to L-serine amino acid-based strategy is paramount for enhancing Sphingolipid content in the host *via de novo* pathways. Our unpublished data demonstrated anti-mycobacterial potential of L-Serine amino acid and supported our hypothesis that such approach may enhance sphingolipids derivatives and may help host in clearing the bacterial burden. This approach would enhance the generation of L-serine rich lipids which promote the differentiation and inflammatory responses of macrophages. Compelling evidences suggest that L-serine enhances the glucose consumption *via* glycolytic pathways which iNOS+ M-1 macrophages need for the optimum immune defense during microbial challenge (16). A recent study has demonstrated that L-serine promotes the synthesis of ceramide and S-1P in mouse embryonic fibroblasts (MEFs) suggesting the important of L-serine in the synthesis of cytoprotective serine rich lipids (17) which are potent activators of resting macrophage to their effector phenotype (18). Interestingly, L-serine bound sphingolipids promote the proliferation of T and B cells (19) and enhance antibody production contributing to Antibody dependent Cell mediated Cytotoxicity (ADCC) which are pre requisite for clearance of both acute and latent infection.

Enhancing Acid Sphingomyelinase (SMAse) activity is another avenue of enhancing host sphingolipids in host because acid sphingomyelinase is essential for several intricate cell signaling pathways (20). ASM binds to the pro-neurotrophin receptor known as sortilin, which is required for *M. tuberculosis* uptake by macrophages (21). Sortilin facilitates the transport of ASM from the Golgi complex into mycobacteria-containing phagolysosomes. Once delivered to the phagolysosomes, acid sphingomyelinase (ASM) associates with lysosomal-associated membrane protein (LAMP) 2 to limit *M. tuberculosis* replication and their subsequent elimination by Bone Marrow Derived Macrophages (BMDM). Furthermore, depleting ASM with the pharmacological inhibitor desipramine increases *M. tuberculosis* survival (21) in LAMP-2+/Voltage-dependent anion channels (VDAC+) phagolysosomes. ASM can also eliminate Bacillus Calmette–Guérin (BCG) effectively by activating reactive oxygen species (ROS) *via* NADPH oxidase subunit p47phox in macrophages. Cathepsin D promotes BCG degradation by ROS in LAMP-2+ compartment. A recent study has demonstrated that acid sphingomyelinase deficiency in macrophages predispose them sensitive for the mycobacterial infection. Adoptive transfer of Wild type macrophages into mice lacking acid-sphingomyelinase completely restored immune defense potential of acid-sphingomyelinase-deficient mice and their susceptibility to BCG. These findings suggest that the acid sphingomyelinase-ceramide system is important for managing mycobacterial infection (20). On the basis of these data, it is likely that by enhancing sphingomyelinase activity in host would in principle elevate Ceramide and reprogram macrophage and promote maturation and acidification of phagolysosomes for eliminating mycobacterial burden. We also expect that sphingomyelinase mediated release of ceramide would get converted to S1P in presence of ceramidase/Sphk-1 and would facilitate M1 differentiation of infected macrophages (12) having Th1 programming capacity in host for reducing mycobacterial load. Both of these strategies (**Figure 1**) hold potential in controlling active TB disease which are low with sphingolipids contents and insensitive to 1^st^ Generation anti TB drugs.

**Figure 1:**
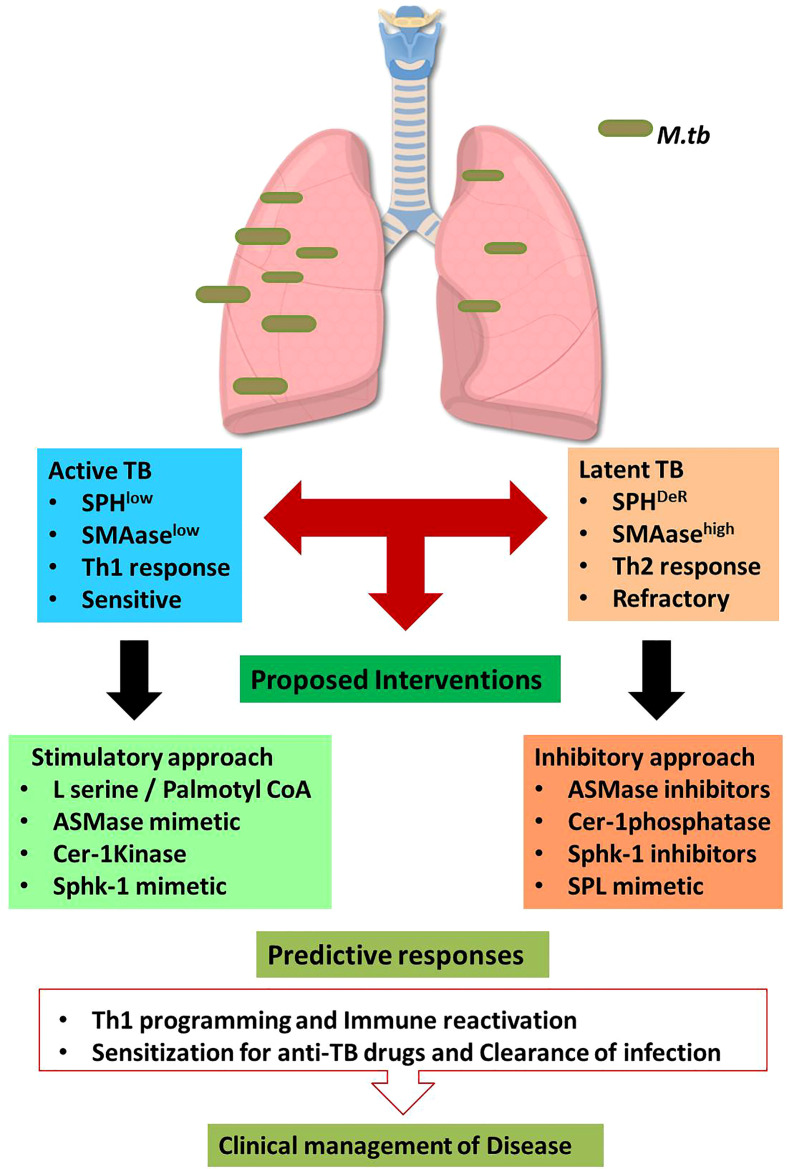
Sphingolipid based approaches for managing tuberculosis.The proposed hypothesis suggests the dual impact of sphingolipids on active and latent tuberculosis. During Active TB, sphingolipids are consumed therefore boosting sphingolipids by indicated interventions may favor host in clearing the bacterial burden. On contrast, latent TB is characterized by accumulation of distinct Sphingolipids derivatives which are involved in refractoriness of host. Thus in such conditions, mitigating sphingolipid levels would augment protective immunity in host. Such approach is anticipated to enhance the sensitivity of patients for 1^st^ Gen anti-TB drugs. This approach is translationally feasible and warrant intense investigation.

However similar approach may not be feasible for managing latent TB cases which, in contrast to active cases, have deregulated sphingolipid/prostaglandins rheostat in their alveolar compartment including foamy macrophages which promote Th2 dominating micromilieu by promoting Th2 programming of effector T cells in the infected lungs (22). This is anticipated to be due to cross regulation of S1PR1 and 2 with EP2/4 receptor associated Cyclooxygenase (COX)-1/2 pathways during chronic and sterile inflammatory responses. Therefore in such latent cases, co-targeting of Sphingolipids alone on in combination of prostaglandins is expected to afford help to host for reducing bacterial load. This is most intricate aspect of TB pathobiology and warrant further in depth investigation for materializing the concept.

The concept proposed in this study is translationally feasible and have tremendous therapeutic potential for managing Tuberculosis.

## Author contributions

Conceptualization, supervision, writing and proof reading, HP. Writing, SM. Editing, RS, AJ, and AS. All authors contributed to the article and approved the submitted version.

## Funding

The work is supported by the funding from Department of Health Research , Govt of India to AS and HP. Grant number - R11013/06/21-GIA-HR.

## Conflict of interest

The authors declare that the research was conducted in the absence of any commercial or financial relationships that could be construed as a potential conflict of interest.

## Publisher’s note

All claims expressed in this article are solely those of the authors and do not necessarily represent those of their affiliated organizations, or those of the publisher, the editors and the reviewers. Any product that may be evaluated in this article, or claim that may be made by its manufacturer, is not guaranteed or endorsed by the publisher.
